# Impact of hybrid FDG-PET/CT on gross tumor volume definition of cervical esophageal cancer: reducing interobserver variation

**DOI:** 10.1093/jrr/rrz004

**Published:** 2019-03-13

**Authors:** Ryo Toya, Tomohiko Matsuyama, Tetsuo Saito, Masanori Imuta, Shinya Shiraishi, Yoshiyuki Fukugawa, Ayumi Iyama, Takahiro Watakabe, Fumi Sakamoto, Noriko Tsuda, Yoshinobu Shimohigashi, Yudai Kai, Ryuji Murakami, Yasuyuki Yamashita, Natsuo Oya

**Affiliations:** 1 Department of Radiation Oncology, Faculty of Life Sciences, Kumamoto University, Kumamoto, Japan; 2 Department of Diagnostic Radiology, Faculty of Life Sciences, Kumamoto University, Kumamoto, Japan; 3 Department of Radiological Technology, Kumamoto University Hospital, Kumamoto, Japan; 4 Department of Medical Imaging, Faculty of Life Sciences, Kumamoto University, Kumamoto, Japan

**Keywords:** positron emission tomography, radiation therapy, esophageal cancer, gross tumor volume, radiotherapy planning, intensity-modulated radiation therapy

## Abstract

Intensity-modulated radiation therapy is being increasingly used to treat cervical esophageal cancer (CEC); however, delineating the gross tumor volume (GTV) accurately is essential for its successful treatment. The use of computed tomography (CT) images to determine the GTV produces a large degree of interobserver variation. In this study, we evaluated whether the use of [^18^F]-fluoro-2-deoxy-D-glucose positron emission tomography (FDG-PET)/CT fused images reduced interobserver variation, compared with CT images alone, to determine the GTV in patients with CEC. FDG-PET/CT scans were obtained for 10 patients with CEC, imaged positioned on a flat tabletop with a pillow. Five radiation oncologists independently defined the GTV for the primary tumors using routine clinical data; they contoured the GTV based on CT images (GTV_CT_), followed by contouring based on FDG-PET/CT fused images (GTV_PET/CT_). To determine the geometric observer variation, we calculated the conformality index (CI) from the ratio of the intersection of the GTVs to their union. The interobserver CI was compared using Wilcoxon’s signed rank test. The mean (±SD) interobserver CIs of GTV_CT_ and GTV_PET/CT_ were 0.39 ± 0.15 and 0.58 ± 0.10, respectively (*P* = 0.005). Our results suggested that FDG-PET/CT images reduced interobserver variation when determining the GTV in patients with CEC. FDG-PET/CT may increase the consistency of the radiographically determined GTV in patients with CEC.

## INTRODUCTION

Cervical esophageal carcinoma (CEC) is an aggressive tumor that arises in the short segment of the esophagus between the cricopharyngeus and the sternal notch. Organ preservation is a common treatment goal for CEC, and radiotherapy (RT), with or without chemotherapy, is the accepted standard of care [1, 2]. RT planning for CEC is challenging. The cervical esophagus lies in close proximity to the spinal cord and courses through the lower neck and upper thorax with drastic changes in contour and diameter of the anatomy. Unlike esophageal cancer arising from other subsites, higher RT doses of 60–66 Gy are usually prescribed for CEC treatment [2, 3], but it is difficult to achieve uniform tumor coverage with 3D conformal RT [4]. Intensity-modulated radiation therapy (IMRT), a more modern technique, allows dose escalation to the levels required to improve local control without increasing the toxicity to normal tissues; this RT technique is being increasingly used to improve locoregional control and survival outcomes in patients with CEC [5, 6]. Meanwhile, the rapid dose drop-off beyond target volumes, which characterizes IMRT, makes treatment success highly dependent on the accurate determination of the gross tumor volume (GTV).

The RT planning process for CEC requires delineating the GTV based on images of a computed tomography (CT) simulator; however, it is difficult to differentiate between tumor and normal tissues using CT images alone because these images provide only morphologic information and poor information pertaining to soft tissues. Furthermore, unlike esophageal cancer of other subsites, physicians can obtain little information on CEC endoscopically; neither endoscopic ultrasound nor clipping are available, even in cases where the tumor is grossly recognized by the endoscope. Therefore, a large degree of interobserver variation exists in defining the GTV during CT image–based planning for CEC [7, 8].

Functional imaging with [^18^F]-fluoro-2-deoxy-D-glucose (FDG)-positron emission tomography (PET) provides information on glucose metabolism and can help manage patients with esophageal cancer for staging and predicting treatment outcomes [9]. FDG-PET/CT fused imaging combines the morphologic information of CT and the metabolic information of PET [10]. Past reports indicated that FDG-PET/CT images reduced observer variation in defining the GTV in patients with esophageal cancer; however, these investigations focused mainly on cancers of the thoracic and/or gastroesophageal junction [11–15], and the usefulness of FDG-PET/CT in defining the GTV in patients with CEC remains unclear. We evaluated whether the use of FDG-PET/CT fused images reduced interobserver variations in determining the GTV, compared with the use of CT images alone, in patients with CEC.

## MATERIALS AND METHODS

### Study population

This retrospective study was approved by the institutional review board of our hospital. Between June 2006 and February 2010, 26 patients with CEC were treated with RT in our hospital. Of these, 11 patients underwent pretreatment FDG-PET/CT scans on a flat tabletop. One patient whose primary tumors were adjacent to the metastatic lymph nodes was excluded to simplify the process of analyzing the GTV in primary tumors exhibited by patients with CEC. Hence, the final study population consisted of 10 patients, including 9 males and 1 female (mean age 64 and range 48–76 years). All patients had pathologically confirmed squamous cell carcinoma, and none of them had previously undergone head-and-neck or thoracic surgery. Prior informed consent was obtained from all patients for the use of their images in future studies.

Patients were restaged according to the TNM classification of malignant tumors (8th edition) promulgated by the Union Internationale Contre le Cancer based on the following examinations: physical examination, endoscopy, barium esophagography, contrast-enhanced whole-body CT, and whole-body FDG-PET/CT. Overall, 8 patients had T3 and 2 had T4 tumors; 6 patients had extensions of the primary lesion beyond the cervical esophagus: hypopharyngeal invasion superiorly in 3 patients and upper-thoracic esophageal invasion inferiorly in 3 patients. Nodal involvement was N0 in 2, N1 in 5, and N2 in 3 patients. Two patients were classified as M1 because of supraclavicular lymph node metastases, and the other 8 patients had no distant metastasis.

### FDG-PET/CT fused imaging

FDG-PET/CT images were obtained using a 3D PET/CT scanner (Gemini GXL 16; Philips Medical Systems, Cleveland, OH, USA). All patients fasted for at least 6 h before the imaging procedure and underwent two routine whole-body PET/CT scans in a single session [at 60–90 min (early scan) and 120–150 min (delayed scan) after receiving an injection of FDG (185–370 MBq)]. In the early scan, we acquired CT images [63 mA, 120 kV, 512 × 512 matrix, 600-mm field of view (FOV), 5-mm slice thickness] and then performed emission measurements in 3D mode with a 144 × 144 matrix. The emission scan time per bed position was 2 min; 10–12 bed positions (FOV 576 mm) were acquired. Attenuation correction was with CT transmission data, and emission images were reconstructed using the line of response–row–action maximum likelihood algorithm; the reconstructed images had a 4-mm slice thickness. The emission scan time per bed position of the delayed scan was 3 min; 4–6 bed positions were acquired. We used a flat tabletop and a pillow dedicated for CT simulation. The patients’ arms were not elevated above their heads. Other imaging protocols were the same as those of the early scan. PET images and CT images for attenuation correction acquired for the delayed scan were transferred to the 3D-radiotherapy planning system (RTPS, Pinnacle^3^ 9.10; Philips Medical Systems, Fitchburg, MA, USA). Registration was performed with RTPS by hardware arrangement.

### GTV definition

The GTVs of all primary tumors were defined by five independent experienced observers: radiation oncologists with an experience of 6–15 years. The observers were provided with routine clinical data (i.e. contrast-enhanced CT, barium esophagogram, and endoscopy reports) and asked to contour the GTV of each primary tumor on axial slices of the CT datasets. For each case, the observers defined the GTV first on the CT image datasets (GTV_CT_), followed by the PET/CT fused image datasets (GTV_PET/CT_). When contouring based on the CT image datasets, observers could not view the PET images. When contouring based on the PET/CT fused image datasets, observers could view the CT images, PET images, and the co-registered PET/CT images. All PET and CT images were initially set to a common window level and width; the observers were allowed to adjust the window level and width to suit their own preferences for contouring on both image datasets.

### Evaluation of intermodality differences and observer variation

For each of the 10 cases, 10 sets of GTVs were defined: 5 (observers) × 2 (modalities). The GTV_CT_ and GTV_PET/CT_ values defined by five observers were calculated to evaluate intermodality (CT-alone images vs PET/CT fused images) differences in the GTVs. For geometric interobserver comparisons, we calculated the conformality index (CI), defined as the ratio of the intersection (A ∩ B) of the GTVs assigned by five observers to their union (A ∪ B); thus, CI = (A ∩ B)/(A ∪ B). The CIs ranged from 0 (complete disagreement) to 1 (perfect concordance); for example, if two volumes overlapped by 50% each, the CI was 0.33 [12, 16].

### Statistical analysis

Intermodality differences in the GTVs and interobserver CIs were compared using the Wilcoxon signed rank test. Statistical calculations were performed with SPSS software, version 24.0 (IBM, Armonk, NY, USA). Differences with *P*-values of <0.05 were considered statistically significant.

## RESULTS

FDG-PET/CT fused images revealed all 10 primary tumors with FDG accumulation. In three patients (30%), an endoscope could not be used because of stenotic tumors.

**Fig. 1.**
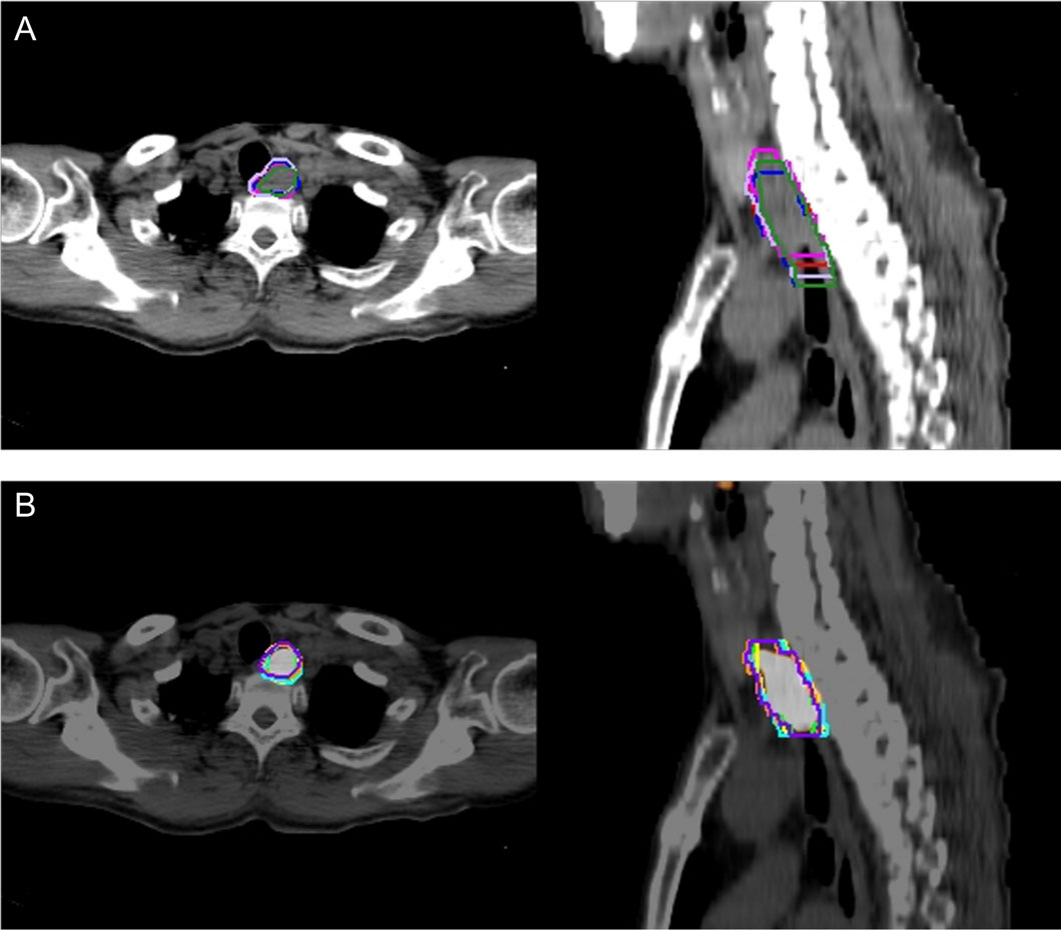
Determining the gross tumor volume (GTV) in a patient with cervical esophageal cancer (cT3N0M0). Each panel includes an axial (left) and a sagittal (right) image. (A) GTVs defined by five observers based on computed tomography (CT)-alone images. The conformality index (CI) was 0.41. (B) The CI improved to 0.59 with positron emission tomography (PET)/CT images.

The mean values of GTV_CT_ and GTV_PET/CT_ were 30.3 cm^3^and 22.9 cm^3^, respectively, for the five observers; GTV_PET/CT_ was significantly smaller than GTV_CT_ (*P* < 0.001, Table 1). The mean interobserver CIs of GTV_CT_ and GTV_PET/CT_ were 0.39 and 0.58, respectively; the interobserver CI of GTV_PET/CT_ was significantly higher than that of GTV_CT_ (*P* = 0.005, Table 2). Figure 1 shows a typical case with the CI of the GTV_PET/CT_ being higher than that of the GTV_CT_.

**Table 1.** Values of GTV_CT_ and GTV_PET/CT_ of 10 patients with cervical esophageal cancer

	Mean ± SD (cm^3^)	Range (cm^3^)	*P*-value*
GTV_CT_	30.3 ± 15.7	1.8–64.5	<0.001
GTV_PET/CT_	22.9 ± 12.8	2.8–51.3	

SD = standard deviation, GTV_CT_ = gross tumor volume defined based on CT-alone images, GTV_PET/CT_ = gross tumor volume defined based on FDG-PET/CT fused images.

*Wilcoxon signed rank test between GTV_CT_ and GTV_PET/CT_

**Table 2.** Interobserver conformality index for GTV_CT_ and GTV_PET/CT_ of 10 patients with cervical esophageal cancer

	Mean ± SD	Range	*P*-value*
GTV_CT_	0.39 ± 0.15	0.05–0.59	0.005
GTV_PET/CT_	0.58 ± 0.10	0.38–0.71	

SD = standard deviation, GTV_CT_ = gross tumor volume defined based on CT-alone images, GTV_PET/CT_ = gross tumor volume defined based on FDG-PET/CT fused images.

*Wilcoxon signed rank test between GTV_CT_ and GTV_PET/CT_

## DISCUSSION

The results of our study suggested that FDG-PET/CT images reduced interobserver variation in GTV determination compared with CT images alone in patients with CEC. A greater consistency in defining the GTV with FDG-PET/CT should reduce the influence of potential variability during RT planning.

The CIs reported previously to determine the primary GTV, using FDG-PET/CT in patients with esophageal cancer, were varied. Vesprini *et al.* evaluated observer variation of six radiation oncologists, with 10 patients with thoracic or abdominal esophageal cancer [11]. The mean CT-estimated GTV was 76 cm^3^. The mean interobserver CIs of the GTV_CT_ and the GTV_PET/CT_ were 0.69 and 0.73, respectively. Wang *et al.* investigated the optimal threshold value for GTV delineation in 12 patients with thoracic esophageal cancer using 4D FDG-PET/CT images [14]. The mean volume of the tumor, estimated based on CT, was 29.4 cm^3^. The best CI between the GTV_CT_ and the PET-based GTV was 0.58 at 20% of the maximum standardized uptake value (SUV_max_). Similarly, Yu *et al.* investigated the optimal threshold value for GTV delineation by FDG-PET/CT, based on the pathological volume (GTV_path_) [17]. Among the 16 patients with a mean GTV_path_ of 28.2 cm^3^, the best CI between the GTV_CT_ and the GTV_path_ was 0.52 at 20% of SUV_max_ or SUV of 2.5. Smaller tumor volumes might have resulted in the lower CIs, and the CI variations found in previous reports could have been due to variable tumor volumes. In this study, five observers contoured relatively small tumor volumes, and our CI results were acceptable in the light of these previous reports.

Previous studies found that combining FDG-PET images with CT images led to smaller GTVs being defined in patients with esophageal cancer. Gondi *et al.* evaluated the effects of adding FDG-PET images to CT images to define the GTV in patients with esophageal cancer [12]. They found that the GTV was reduced by >5% in 10 of 16 (62.5%) patients. Muijs *et al.* evaluated the tumor length in 21 patients with esophageal cancer [13]. The addition of PET changed the average tumor length significantly, from 73 mm on CT to 65 mm on PET/CT (*P* = 0.02). They also found that combining PET and CT images led to a decreased tumor length in 11 of 21 (52%) patients, with a mean reduction of 17 mm. On the other hand, in 5 (24%) patients, there was a mean increase of 6 mm. A trend toward reduced tumor sizes when combining PET and CT images was found in our study.

There were some limitations to our study. First, we retrospectively evaluated the effect of adding PET images when defining the GTV in patients with CEC, but we did not evaluate the effect of adding PET images to dose distributions or treatment outcomes. Second, we used CT images for attenuation correction because PET/CT scans for the simulation were not obtained for all patients. Relatively lower image quality of CT images than those for the simulation may have affected our results. Third, as we evaluated the value of PET/CT for GTV definition based on the FDG-avid diseases of T3 or T4 patients, we are unable to comment on the cases of non-FDG-avid early or superficially extended lesions. Further prospective clinical trials based on PET/CT simulation will be required in order to evaluate the clinical benefits for patients undergoing treatment for CEC.

In conclusion, the use of FDG-PET/CT images appears to reduce interobserver variations when determining the GTV in patients with CEC. FDG-PET/CT may improve the consistency of pre-RT planning for patients with CEC.
